# Single-cell RNA-seq reveals *FGF12* as a prognostic biomarker in low-grade endometrial stromal sarcoma

**DOI:** 10.3389/fimmu.2024.1513076

**Published:** 2024-11-29

**Authors:** Yu Miao, Meng Dong, Qiyin Zhou, Julia Thiel, Na Li, Ying Cai, Dan Yuan, Haitao Wang, Su-Han Jin, Hua Yang, Jinjing Wang, Benjamin Frey, Udo S. Gaipl, Hu Ma, Jian-Guo Zhou

**Affiliations:** ^1^ Department of Oncology, The Second Affiliated Hospital of Zunyi Medical University, Zunyi, China; ^2^ Institute of Biomedical Research, Henan Academy of Sciences, Zhengzhou, China; ^3^ Dr. Margarete Fischer-Bosch Institute of Clinical Pharmacology and University of Tübingen, Stuttgart, Germany; ^4^ Department of Obstetrics and Gynecology, Yanhe County People’s Hospital, Tongren, Guizhou, China; ^5^ Department of Obstetrics and Gynecology, The Second Affiliated Hospital of Zunyi Medical University, Zunyi, Guizhou, China; ^6^ Department of Pathology, The Affiliated Hospital of Zunyi Medical University, Zunyi, China; ^7^ Thoracic Surgery Branch, Center for Cancer Research, National Cancer Institute (NCI), NIH, Bethesda, MD, United States; ^8^ Department of Orthodontics, Affiliated Stomatological Hospital of Zunyi Medical University, Zunyi, China; ^9^ Department of Pathology, The Second Affiliated Hospital of Zunyi Medical University, Zunyi, China; ^10^ Translational Radiobiology, Department of Radiation Oncology, Universitätsklinikum Erlangen, Friedrich-Alexander-Universität Erlangen-Nürnberg, Erlangen, Germany; ^11^ Comprehensive Cancer Center Erlangen, European Metropolitan Region Nuremberg, Erlangen, Germany; ^12^ FAU Profile Center for Immunomedicine, Friedrich-Alexander-Universität Erlangen-Nürnberg, Erlangen, Germany

**Keywords:** scRNA-seq, *FGF12*, low-grade endometrial stromal sarcoma, tumor microenvironments, prognostic biomarker

## Abstract

**Background:**

Low-grade endometrial stromal sarcoma (LG-ESS) is a rare uterine malignancy characterized by its complex tumor microenvironment (TME) and high recurrence rates, posing challenges to accurate prognosis and effective treatment. Identifying prognostic biomarkers is essential for improving patient stratification and guiding therapeutic strategies.

**Methods:**

Using single-cell transcriptome analysis combined with H&E and multiplex immunofluorescence staining, we identified a subpopulation of tumor cells in LG-ESS and further validated the association of this subpopulation and its characteristic genes with LG-ESS prognosis by molecular characterization and bulk transcriptome data.

**Results:**

Our analysis reveals multiple cellular subpopulations within the tumor tissue, particularly a tumor cell subpopulation among them which is associated with poor prognosis. Originating from normal stromal fibroblasts, this subpopulation appears to play a crucial role in TME remodeling, smooth muscle cell behavior, and potentially in tumorigenesis and metastasis. Of particular interest in this subpopulation is the highly expressed *FGF12* gene, which is significantly associated with a shortened survival in ESS, highlighting its potential as a prognostic biomarker.

**Conclusion:**

Our study reveals the complexity of TME within the LG-ESS and highlights the role that tumor cell subpopulations play in disease progression and patient prognosis. The identification of *FGF12* as a prognostic biomarker suggests a new approach for the personalized treatment and prognosis monitoring of patients.

## Introduction

Endometrial stromal sarcoma (ESS) is a rare uterine malignancy originating from endometrial mesenchymal cells (1), accounting for less than 1% of all uterine tumors (2). ESS was classified by the World Health Organization into four types in 2020 based on clinical and pathologic features and progress in molecular genetic studies: endometrial stromal nodule (ESN), low-grade ESS (LG-ESS), high-grade ESS (HG-ESS), and undifferentiated uterine sarcoma (UUS) (3). Although LG-ESS is usually slow-growing and has a relatively good prognosis (5-year overall survival rate of more than 80%) (4, 5), its recurrence and late mortality rates are still not negligible, approximately 60% and 15%-25%, respectively (6, 7). Due to its variable behavior and limited understanding of its pathobiology, LG-ESS represents a significant therapeutic challenge (4, 8, 9). In addition, the lack of distinctive clinical manifestations makes preoperative imaging difficult to diagnose, necessitating reliance on postoperative pathologic assessments (10, 11). However, this reliance inadvertently increases the risk of misdiagnosis and underdiagnosis, potentially impacting patient management and prognostic outcomes.

Current therapeutic strategies for LG-ESS generally follow the approaches used for other sarcomas, including surgery, hormonal therapy, radiotherapy, and chemotherapy (4, 12, 13). However, despite the generally favorable prognosis in early-stage cases, LG-ESS tends to recur, and advanced or recurrent disease remains difficult to treat with the current therapeutic options (14–16). The rarity of LG-ESS, coupled with the dearth of detailed insights into its molecular foundations and comprehensive molecular profiling, has impeded the advancement of innovative therapeutic strategies, including targeted therapies that are pivotal in modern precision medicine (17).

Despite advances in the understanding of uterine malignancies, the precise molecular and cellular mechanisms driving LG-ESS remain elusive. While hormone receptor expression (18–20) and the presence of chromosomal rearrangements, such as *JAZF1-SUZ12* and *JAZF1-PHF1* fusions (21), have been implicated in the pathogenesis of LG-ESS, the specific cellular subpopulations responsible for tumor initiation, progression, and recurrence have not been comprehensively characterized. As a result, there is a critical need to delineate the molecular characterization of LG-ESS to identify potential therapeutic vulnerabilities and improve treatment outcomes for patients.

In addition, predicting the clinical course of LG-ESS remains challenging, and the risk of recurrence is high. Currently, prognostic biomarkers for LG-ESS are largely based on clinical and pathological characteristics such as tumor size, stage at diagnosis, and hormone receptor status. While these factors provide some predictive value, they are insufficient for accurately identifying patients at high risk for recurrence or poor prognosis. Identifying molecular markers that can accurately predict patient outcomes will not only improve treatment planning but also enhance the overall management of this rare and challenging malignancy. A reliable prognostic biomarker for LG-ESS would allow clinicians to stratify patients into different risk categories, guiding treatment decisions accordingly. Patients with a higher risk of recurrence or poor survival outcomes could benefit from more aggressive treatment strategies, such as adjuvant therapy or closer post-surgical monitoring, while those with a lower risk could avoid unnecessary interventions.

Tumors are not homogenous entities but rather complex ecosystems composed of diverse cell types, with each contributing differently to tumor progression, immune evasion, and therapeutic resistance. For LG-ESS, exploring this heterogeneity is especially critical given the limited understanding of its stromal origin and how the tumor microenvironment may influence its behavior. Advances in single-cell transcriptomics have provided unprecedented insights into the heterogeneity of cellular populations within the tumor immune microenvironment (TME), enabling the identification of subpopulations that may contribute to tumorigenesis, disease progression, and therapeutic resistance (22). The application of these technologies to LG-ESS research holds promise for unveiling the intricate cellular composition and deepening our understanding of the molecular drivers of tumor behavior. The identification of unique molecular signatures in tumor cell subpopulations could lead to the discovery of novel biomarkers and therapeutic targets, potentially revolutionizing the treatment landscape for LG-ESS.

This study aims to characterize LG-ESS using single-cell transcriptomics to describe the cellular composition, molecular characteristics and identify potential prognostic biomarkers. The insights gained from this study are expected to lay the cornerstone for a new paradigm in the personalized treatment of endometrial stromal sarcomas.

## Methods

### Sample collection and sequencing

A sample of LG-ESS was collected immediately after surgery, some of which were fixed with 10% formalin fixation, and some were snap frozen. The formalin fixed sample was further embedded in paraffin for immunohistochemical staining. The snap frozen sample was digested and dispersed to form a single-cell suspension. Single cell capture, reverse transcription, amplification, and library construction were performed using the 10X Genomics platform according to the manufacturer’s instructions. Libraries were then sequenced, and data was processed using CellRanger software to generate a single cell transcriptome data matrix. The study was approved by the Ethics Committee of Zunyi Medical University (No. 2020-1-013), with informed consent obtained from all participants.

### Data collection

Single-cell transcriptome sequencing data for five healthy control (HC) endometrial samples (raw fastq files) were downloaded from the NCBI SRA database, accession number SRP349751 (23). Bulk transcriptomic datasets used for validation were sourced from the GEO database (GSE128630, GSE119041, and GSE85383). The GSE128630 dataset encompasses 75 ESS cases (24). GSE119041 includes 13 leiomyosarcoma (LMS), 16 ESS, 26 UUS, and three YWHAE-FAM22 endometrial stromal sarcomas (YFAM) samples, along with 14 benign leiomyoma controls (LM) (25). GSE85383 contains 9 LG-ESS, 4 HG-ESS, 8 UUS, and 4 uterine LMS (26). The clinical characteristics of these samples were extracted from the corresponding publications.

### Identification and removal of doublets

DoubletFinder (version 2.0.3) (27) was used for each sample to identify and remove doublets, with the doublet rate set to 0.03 for 10x genomic sequencing.

### Data preprocessing

Data integration of Seurat objects after doublet removal was performed using the Seurat (R package,version 4.4.0) (28), filtering out abnormal cells (mito_percent < 15% & nFeature > 200 & nFeature < 6000) and logarithmizing the UMIcount matrix. In order to eliminate experimental batch effects, the harmony package (version 1.0.3) was used for the integration of the data (29).

### Cell type identification

The top 2000 highly variable genes were selected for principal component analysis (PCA), with principal components determined through the JackStraw procedure. After determining the selected number of principal components (dims = 1:30) during the integration process, we clustered the cells with a resolution of 1 and performed dimensionality reduction with t-SNE for visualization. Cell subpopulations were named based on SingleR (version 2.2.0) annotation and classic marker expression (23, 30).

### RNA velocity analysis

We performed RNA velocity analysis based on bam files generated from *CellRanger* Count (version 7.2.0) analysis using the *scVelo* software (version 0.2.5) (31). scVelo infers temporal dynamic changes in gene expression by estimating splicing dynamics and unspliced/spliced ratios of individual cells. Finally, based on the RNA velocity results, we can reveal the dynamic changes in cell status and their potential differentiation pathways in the LG-ESS tumor sample.

### Trajectory analysis

For trajectory analysis, Monocle 2 (version 2.18.0) (32) was used to investigate dynamic changes in cell states based on the expression of variable genes in the target population (cells expressing ≥10 genes with an average expression >0.5). Genes with significant temporal variation were identified using the differentialGeneTest function. Dimensionality reduction was performed with the DDRTree algorithm, and a minimum spanning tree was constructed using the plot_cell_trajectory function. To visualize gene expression changes along pseudotime, the top 100 dynamically expressed genes were clustered using the plot_pseudotime_heatmap function. Functional enrichment analysis for each gene cluster was conducted using KOBAS 3.0.

### Analysis of intercellular communication

In order to investigate the interactions between different cell types in the tumor microenvironment of LG-ESS, we performed intercellular communication analysis using CellPhoneDB2 (database version v2.0.0) (33), a Python-based computational analysis tool, to identify interaction networks.

### Estimation of cell proportions in samples of bulk transcriptome

To quantify the cell type composition within our bulk transcriptome samples, we utilized the BisqueRNA (version 1.0.5) algorithm (34). This tool allowed us to leverage the gene expression profiles derived from identified subpopulations in single-cell RNA sequencing data. Through this deconvolution approach, we were able to infer the relative abundances of different cell types present in the mixed tissue samples.

### Enrichment analysis

We performed gene set enrichment analysis (GSEA) and Kyoto Encyclopedia of Genes and Genomes (KEGG) enrichment analysis on the differential gene expression results using the clusterProfiler (version 4.8.3) (35) and kobas (version 3.0) software, respectively.

### Survival analysis and Cox regression analysis

Survival analysis: We used the cutoff values (median expression or best cutoff value) of the expression of specific genes in the GSE128630 cohort and the GSE119041 cohort to divide the cohort into two groups and used the survival package to compare the differences in survival rates between the groups and to plot Kaplan-Meier (KM) curves.

Univariate Cox regression analysis: Cox regression modeling was performed using the survival package of the R software, and one-way Cox regression was performed with the expression of the gene of interest as a covariate.

Multivariate Cox regression analysis: Cox regression modeling was performed using the survival package in R software, incorporating relevant clinical factors such as age at diagnosis, tumor grade, and BMI, alongside the expression levels of *FGF12* and *KLHL29* as covariates. This approach allowed us to assess the independent prognostic significance of *FGF12* and *KLHL29* while adjusting for the effects of these clinical variables.

### H&E staining

The paraffin-embedded tissue samples (FFPE) are cut into 3-5 micron sections. For H&E staining, the sections are stained in an acid hematoxylin solution for about 8 minutes and then in an eosin solution for about 2.5 minutes using a slide stainer.

### Multiplex immunofluorescence (mIF) staining

A 7-color multiplex immunofluorescence staining was performed using the OPAL™ multiplexing method. The staining protocol for FFPE tissue sections was optimized for the simultaneous detection of 6 antibodies and DAPI as a nuclear stain. The fixed tissues were cut into 4µm sections by Rotary Microtome (Leica RM2255, Germany). The sections were deparaffinized, rehydrated, subjected to heat-induced epitope retrieval and incubated with primary and secondary antibodies. The antibodies were visualized using the fluorescent tyramide from the Opal 6-Plex Manual Detection Kit (NEL861001KT, Akoya Biosciences). The process of epitope retrieval and staining was repeated sequentially for different primary antibodies and fluorescent tyramide combinations. The following primary antibodies with different dilutions were used: CD10 (#110M-16, Cell Marque) with 1:40 dilution, Desmin (#243M-14, Cell Marque) with 1:50 dilution, CD4 (#104R-14, Cell Marque) with 1:100 dilution, CD8 (#108R-14, Cell Marque) with 1:75 dilution, CD68 (#168M-94, Cell Marque) with 1:75 dilution, FOX-P3 (#14-4777-82, eBioscience). Antibodies were visualized with the following tyramide dyes from the Opal Detection kit (NEL861001KT, Akoya Biosciences): Opal Polaris 480, Opal 520, Opal 570, Opal 620, Opal 690, and DIG-Opal 780. Sections were mounted with ProLong^®^ Diamond Antifade Mountant (P36961, Thermo Fisher Scientific). Multiplex-stained slides were imaged using a PhenoImager Fusion system (Akoya Biosciences).

### Statistical analysis

The log-rank test was used for survival analysis. For other intergroup comparisons, the Wilcoxon rank-sum test was used. P<0.05 is considered to be statistically significant.

## Result

### Altered gene expression profiles in multiple subpopulations of LG-ESS

**Figure 1:**
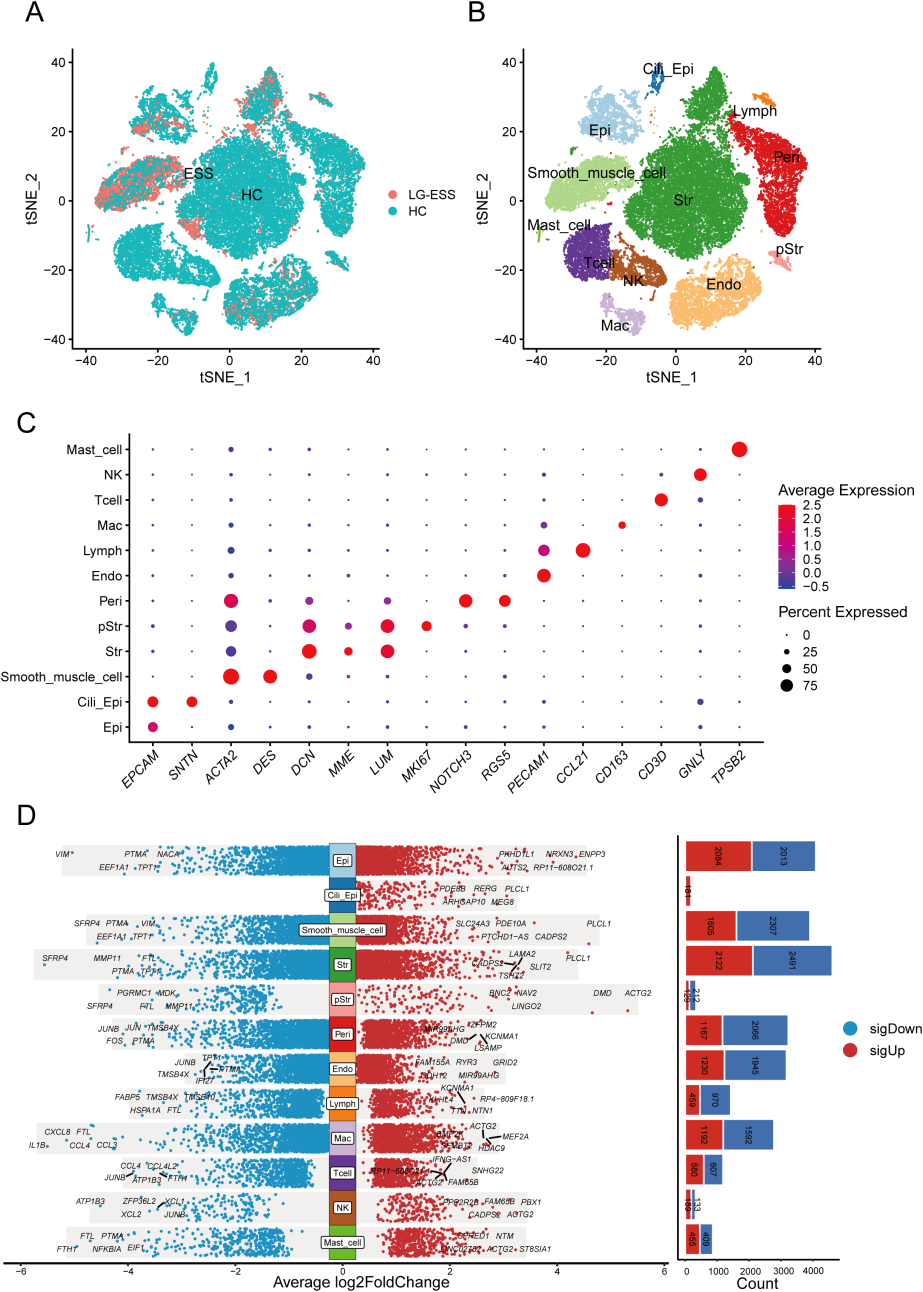
Single-cell transcriptome analysis reveals altered gene expression profiles in multiple cellular subpopulations in LG-ESS. **(A)** Scatterplot showing the overall distribution of cell subpopulations in HC and LG-ESS. **(B)** t-SNE plot showing the major cell types identified from HC and LG-ESS groups in different colors. **(C)** Scatterplot showing the expression of representative marker genes in different cell types. **(D)** Differential genes between different cell subpopulations of HC and LG-ESS, red color indicates genes up-regulated in LG-ESS, blue color indicates genes down-regulated in LG-ESS, and the bar graph on the right side shows the number of differential genes.

To investigate the cellular composition of LG-ESS, we collected a fresh tissue sample from an LG-ESS patient during surgery and performed single-cell transcriptomic sequencing. Concurrently, we rigorously curated and integrated this dataset with single-cell transcriptomic data from five healthy controls (HCs) sourced from a public database, ensuring stringent quality control throughout the analysis (**Figure 1A**).

Following batch correction (**Supplementary Figure S1A**), we dimensionally reduced and clustered 38,288 cells, identifying 12 primary cell subpopulations (**Figure 1B**): non-ciliated epithelial cells (Epi, EPCAM^+^ SNTN^-^), ciliated epithelial cells (Cili_Epi, EPCAM^+^ SNTN^+^), Str (stromal fibroblasts, DCN^+^ MME^+^ LUM^+)^, smooth muscle cells (Smooth_muscle_cell, ACTA2^+)^, proliferative stromal cells (pStr, MKI67^+)^, pericytes (Peri, NOTCH3^+^ RGS5^+)^, endothelial cells (Endo, PECAM1^+)^, lymphatic endothelial cells (Lymph, CCL21^+)^, macrophages (Mac, CD163^+)^, T cells (Tcell, CD3D^+)^, natural killer cells (NK, GNLY^+)^, and mast cells (Mast_cell, TPSB2^+)^ (**Figure 1C**). **Supplementary Figure S1B** displays the top five highly expressed genes in each cell subpopulation, and **Supplementary Figure S1C** shows the quantification of all cell subpopulations from each sample. Furthermore, differential gene analysis of all subpopulations between LG-ESS and HC revealed that multiple subpopulations exhibited significant differences in gene expression, particularly within the Str subpopulation (**Figure 1D**). We speculate that changes in the gene expression profile of this subpopulation may be intricately linked to the pathogenesis of LG-ESS.

### The tumor cell subpopulation, Str1, significantly associated with poor prognosis of LG-ESS

**Figure 2:**
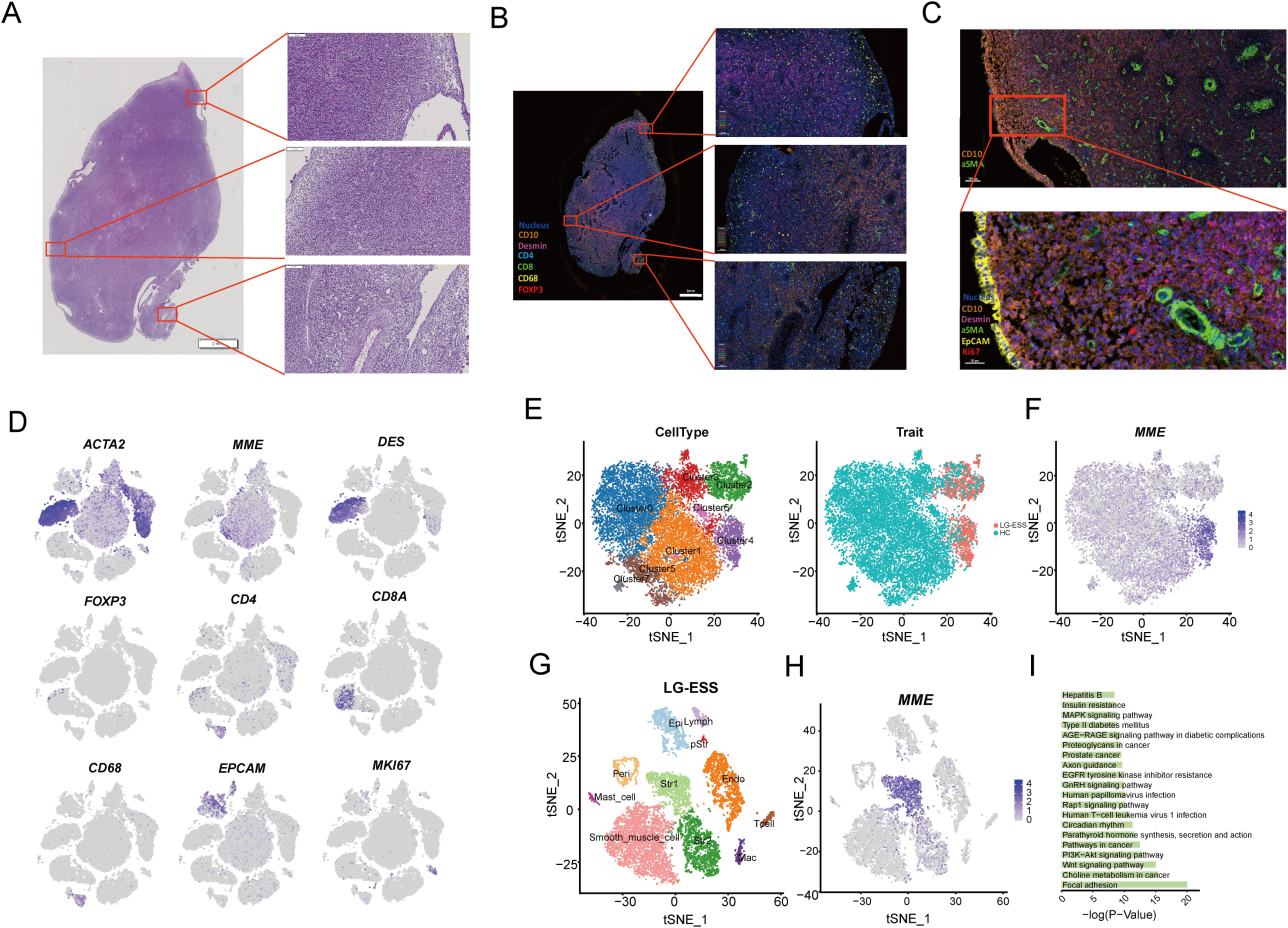
Characterization and functional analysis of LG-ESS tumor cell subpopulations. **(A)** H&E staining result of the LG-ESS tissue sample, highlighting the overall tissue architecture at low magnification, with higher magnification images of selected regions showing cellular morphology. **(B, C)** miF staining result of LG-ESS sample. CD10, desmin, and other markers are used to distinguish different cell populations within the tumor, with magnified views focusing on specific regions of interest. The scale in figure **(B)** represents 2 mm and 100 µm, while in figure **(C)** it represents 100 µm and 30 µm. **(D)**. Expression of key marker genes from the single-cell RNA sequencing data corresponding to the same LG-ESS sample. These markers (e.g., *ACTA2*, *MME*, *DES*, *FOXP3*, *CD4*, *CD8A*) help identify distinct cell types within the tumor microenvironment. **(E)** t-SNE plots showing the clustering of cell subpopulations (left) in the LG-ESS sample and their distribution in HC and LG-ESS tissue (right). **(F)** Featureplot demonstrating the expression of the CD10 (MME) gene in different subgroups of Str. **(G)** t-SNE plot highlighting the main cell types present in LG-ESS, identified by distinct colors. **(H)** MME expression across all cell types in the LG-ESS sample, with Str1 cells showing prominent expression. **(I)** Results of KEGG enrichment analysis of genes highly expressed in Str1.

Previous studies have indicated that most LG-ESS exhibit positive expression of CD10 (protein of MME gene) and WT1, with elevated levels of estrogen receptor expression (20, 36, 37). Through H&E staining (**Figure 2A**) and multiplex immunofluorescence (mIF) staining (**Figures 2B, C**; **Supplementary Figure S2A**) of adjacent tissue sections, we found that the tumor cell-rich regions of the LG-ESS sample displayed positive CD10 expression (**Figure 2B**, right middle) and were located adjacent to smooth muscle cell populations (α-SMA^+^ DES^+^) (**Figure 2B**). Additionally, the tumor sample contained various other cell types, including epithelial cells (EPCAM^+^), pStr (MKI67^+^) CD4^+^T cells (CD4^+^), CD8^+^ T cells (CD8A^+^), macrophages (CD68^+^), and regulatory T cells (FOXP3^+^), which were further validated by our single-cell data (**Figure 2D**). Unlike most epithelial-derived solid tumors (including endometrial carcinoma (22)), LG-ESS originates from mesenchymal fibroblasts rather than endometrial epithelial cells (4). Consistent with this notion, we found that MME^+^(CD10) was predominantly expressed in the Str subpopulation, with almost no expression in epithelial cells.

To explore the functional roles of tumor cell subpopulations in LG-ESS, we re-clustered the Str subpopulation (**Figure 2E**), resulting in eight distinct clusters. We observed that Cluster2 and Cluster4 were primarily composed of LG-ESS, and notably, LG-ESS tumor cells (MME^+^) were mainly located in Cluster4 (**Figure 2F**). Consequently, we independently clustered the LG-ESS samples and further differentiated the Str subpopulation into two subgroups: Str1, characterized by MME^+^ tumor cells (predominantly in Cluster4), and Str2, defined by MME- cells (predominantly in Cluster3) (**Figures 2G, H**). KEGG enrichment analysis indicated that the highly expressed genes in the Str1 tumor cell subgroup were significantly enriched in several classic cancer-related pathways, including focal adhesion, choline metabolism in cancer, Wnt signaling pathway, and PI3K-Akt signaling pathway (**Figure 2I**). This aligns with previous findings demonstrating significant activation of the Wnt signaling pathway in LG-ESS (38).

Subsequently, we utilized BisqueRNA to estimate the proportions of cells in a large number of transcriptomic samples based on the top 50 most highly expressed genes across all LG-ESS cell subpopulations, and we explored the relationship between subpopulation proportions and the prognosis of LG-ESS. We found that an increased proportion of Str1 in LG-ESS was significantly associated with shorter overall survival and progression-free survival of patients (**Supplementary Figure S2B**). At the same time, we observed no significant differences in the proportion of Str1 across different sarcoma types (**Supplementary Figure S2C**).

### Tumor cells in LG-ESS derive from normal stromal fibroblasts rather than MME^-^ Str subpopulation

**Figure 3:**
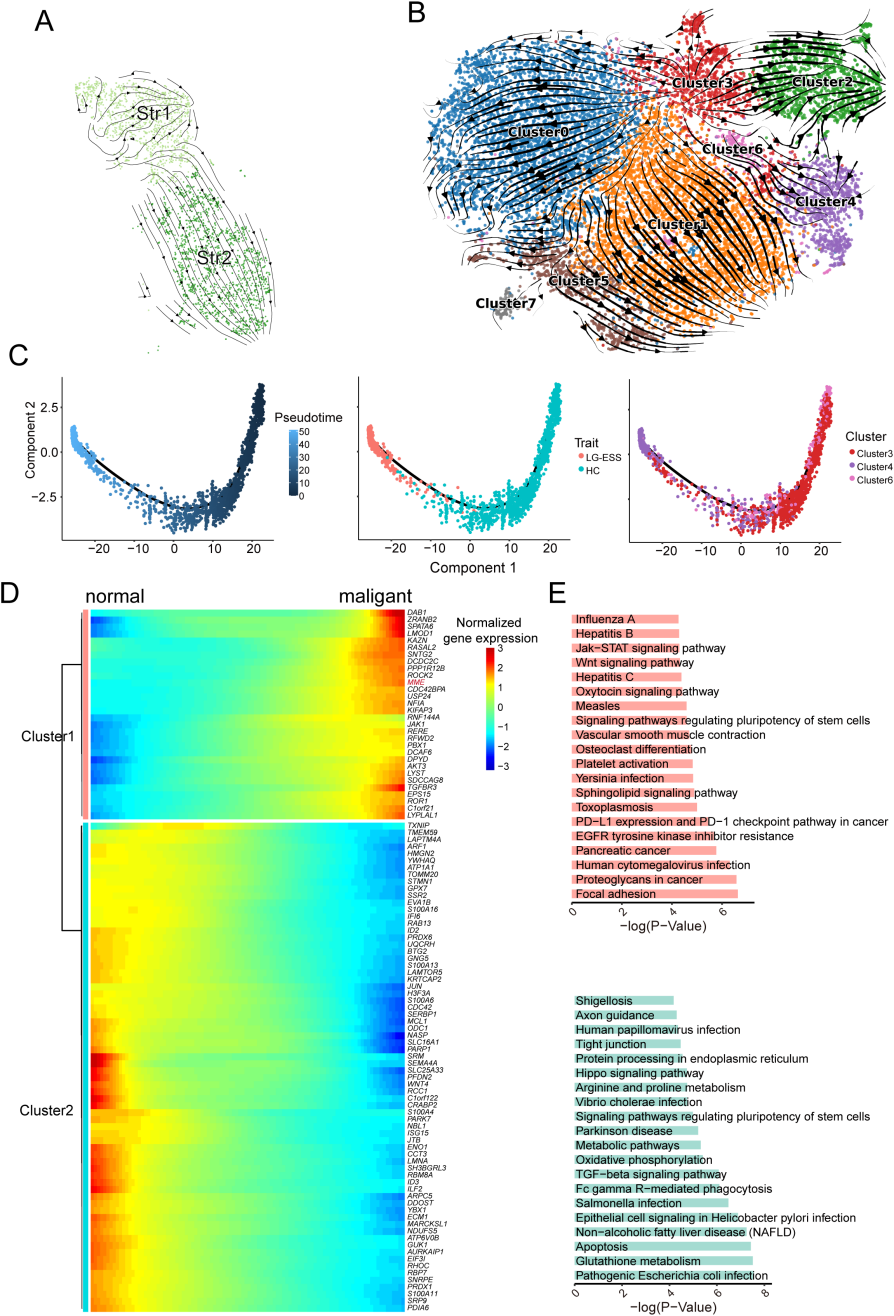
Origin of Str1 tumor cell subpopulations in LG-ESS. **(A)** RNA velocity analysis of Str1 and Str2 subpopulations in LG-ESS, illustrating their potential shared ancestral origin. **(B)** RNA velocity analysis of Str subpopulations from HC and LG-ESS samples, showing lineage relationships between clusters. **(C)** Pseudotime analysis of Cluster3, 4, and 6, demonstrating differentiation paths in Str cell subpopulations. **(D)** Heatmap of gene expression changes during the differentiation of normal stromal fibroblasts to malignant cells. **(E)** Results of KEGG enrichment analysis of significantly upregulated (top) and downregulated (below)pathways during tumor cell differentiation.

To further elucidate the origins of the Str1 tumor cell subpopulation in LG-ESS, we conducted RNA velocity analysis on both Str1 and Str2 subpopulations to determine their lineage relationships. Surprisingly, our findings indicated that Str1 and Str2 may share a common ancestral origin, contradicting our initial hypothesis that Str1 arose from Str2 (**Figure 3A**).

To validate this finding, we utilized the Str subpopulation from HC as a normal reference and performed RNA velocity analysis on all Str cells from both HC and LG-ESS samples. We discovered that Str1 (predominantly corresponding to Cluster4) and Str2 (primarily corresponding to Cluster2) indeed originated from the same ancestral cluster, Cluster3. Moreover, Cluster4 originating from Cluster3 through the intermediate Cluster6 (**Figure 3B**). Simultaneously, we conducted pseudo-time analysis on Clusters3, Clusters4, and Clusters6 using Monocle2. Consistent with the RNA velocity analysis, our results confirmed that Cluster4, which primarily comprises the Str1 tumor cell subpopulation in LG-ESS, predominantly develops from Cluster3 and Cluster6 (**Figure 3C**).

To explore the characteristic changes occurring during the differentiation of tumor cells, we clustered the genes that exhibited significant changes throughout the differentiation process (**Figure 3D**). KEGG enrichment analysis revealed that genes that were significantly upregulated during differentiation were primarily associated with common cancer-related pathways, such as focal adhesion, proteoglycans in cancer, Wnt signaling pathway, and Jak-STAT signaling pathway. Conversely, genes that were significantly downregulated during differentiation were predominantly linked to metabolic processes and oxidative phosphorylation pathways (**Figure 3E**).

### High expression of *FGF12* is significantly associated with poor prognosis of LG-ESS

**Figure 4:**
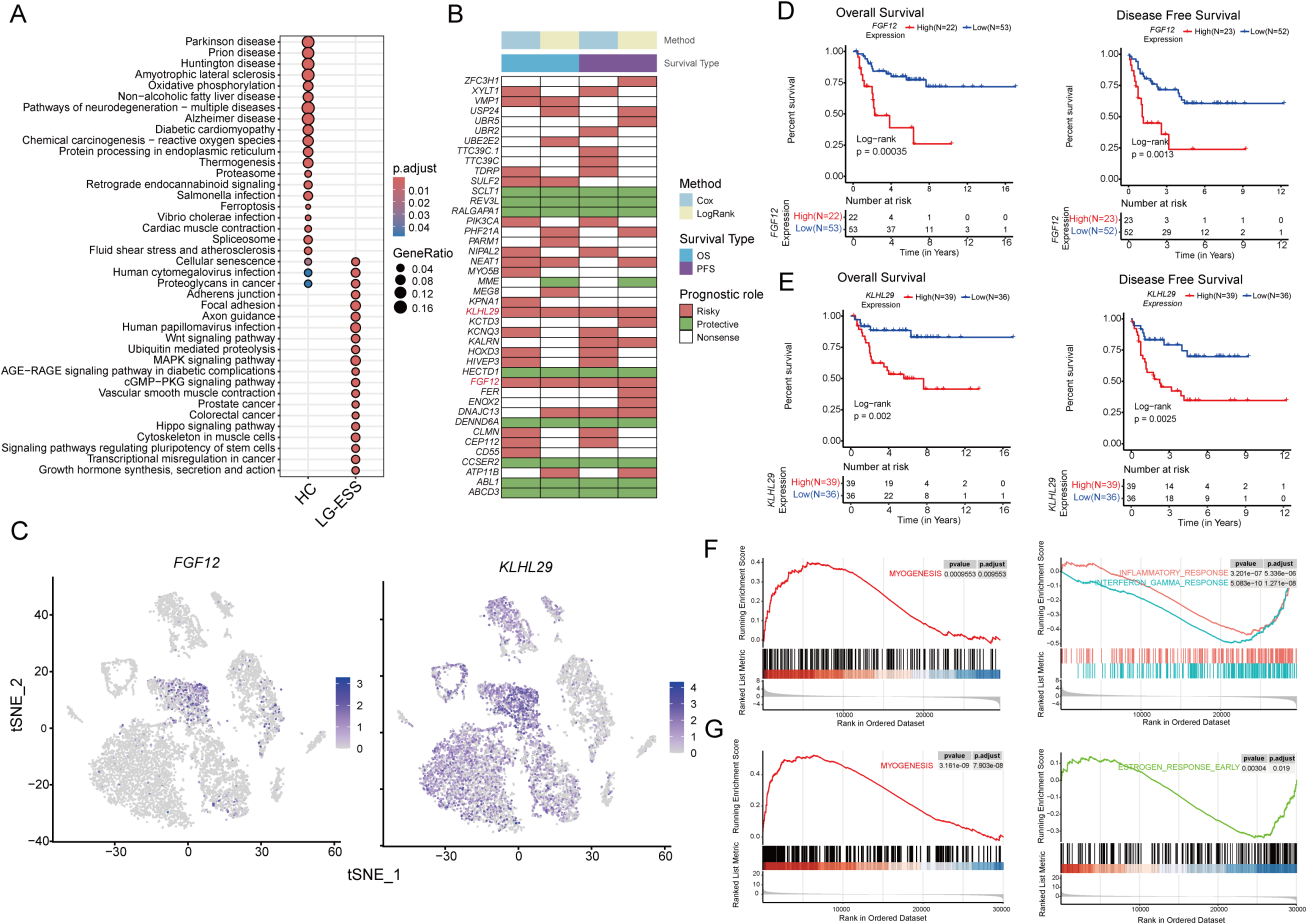
*FGF12* and *KLHL29* expression correlates with poor prognosis and tumor progression in ESS. **(A)** Scatterplot demonstrating the results of KEGG enrichment analysis of differentially expressed genes of Str1 between LG-ESS and HC. **(B)** Heatmap demonstrating the results of Cox regression analysis and survival analysis for specific genes based on bulk transcriptome data of ESS patients. **(C)** Featureplot demonstrating the expression of *FGF12* and *KLHL29* genes in different cellular subpopulations of LG-ESS. **(D, E)** Results of survival analysis based on *FGF12* **(D)** and *KLHL29* **(E)** expression in ESS patients, with overall survival on the left and progression-free survival on the right (GSE128630). **(F, G)** Results of GSEA enrichment analysis after differentiating patients into high and low expression groups based on *FGF12* **(F)** and *KLHL29* **(G)** gene expression in ESS patients, with significantly activated pathways in the high expression group on the left and significantly inhibited pathways on the right.

Upon investigating the biological alterations within tumor cell subpopulations of ESS as compared to HC, our enrichment analyses of differentially expressed genes (LG-ESS *vs.* HC) within the Str-Cluster4 subpopulation (**Figure 2E**) indicated significant gene expression modifications. These modifications predominantly clustered around pathways integral to oncogenesis and tumor progression, including growth hormone synthesis, secretion, and action, along with cell cycle regulation, focal adhesion, Wnt, and MAPK signaling pathways (**Figure 4A**). To investigate the underlying drivers of these pathways, we performed Cox regression and survival analysis on these significantly differentially expressed genes based on the gene expression data of ESS patients from the GEO database, focusing on genes uniquely expressed in Str1 (**Figure 4B**). Notably, high expression of *FGF12* and *KLHL29* in ESS was significantly associated with shorter overall and progression-free survival (**Figures 4C-E**). Through multivariate cox regression analysis, we found that FGF12 and KLHL29 remain important independent prognostic factors affecting overall survival (**Supplementary Figure S3A**) and progression-free survival (**Supplementary Figure S3B**). In addition, an independent cohort of patients further validated the association between high *FGF12* gene expression and shortened overall survival (OS) in uterine sarcoma patients (**Supplementary Figure S3C**), suggesting that *FGF12* could potentially be a prognostic biomarker for ESS.

Moreover, a comparative analysis of *FGF12* and *KLHL29* expression across different sarcoma subtypes and leiomyosarcomas (LMS) revealed a significant difference in *KLHL29* expression between HG-ESS and LG-ESS and LMS. In contrast, *FGF12* expression showed no significant difference. This indicates that *KLHL29* may serve as a potential biomarker for distinguishing HG-ESS from LG-ESS (**Supplementary Figure S3D**).

To further elucidate the biological functions of *FGF12* and *KLHL29* in ESS, we categorized ESS samples into high- and low-expression groups based on their gene expression levels and conducted GSEA enrichment analysis. We observed that the MYOGENESIS pathway was significantly activated in both the *FGF12* ^high^ and *KLHL29* ^high^ groups. Conversely, immune-related pathways, such as the INFLAMMATORY RESPONSE and INTERFERON GAMMA RESPONSE, were notably suppressed in the *FGF12* ^high^ group, while the ESTROGEN RESPONSE EARLY pathway was significantly suppressed in the *KLHL29* ^high^ group (**Figures 4F, G**).

### Functional aberrations in cell subpopulations influenced by tumor cells in LG-ESS

Previous studies have shown that in epithelial-derived tumors, fibroblasts within the tumor microenvironment may be induced into cancer-associated fibroblasts (CAFs) through interactions with tumor cells (39, 40), and macrophages may undergo M2 polarization under the influence of tumor-derived factors (41). These phenotypic and functional changes in stromal and immune cells within the tumor microenvironment, in turn, contribute to tumor progression.

**Figure 5:**
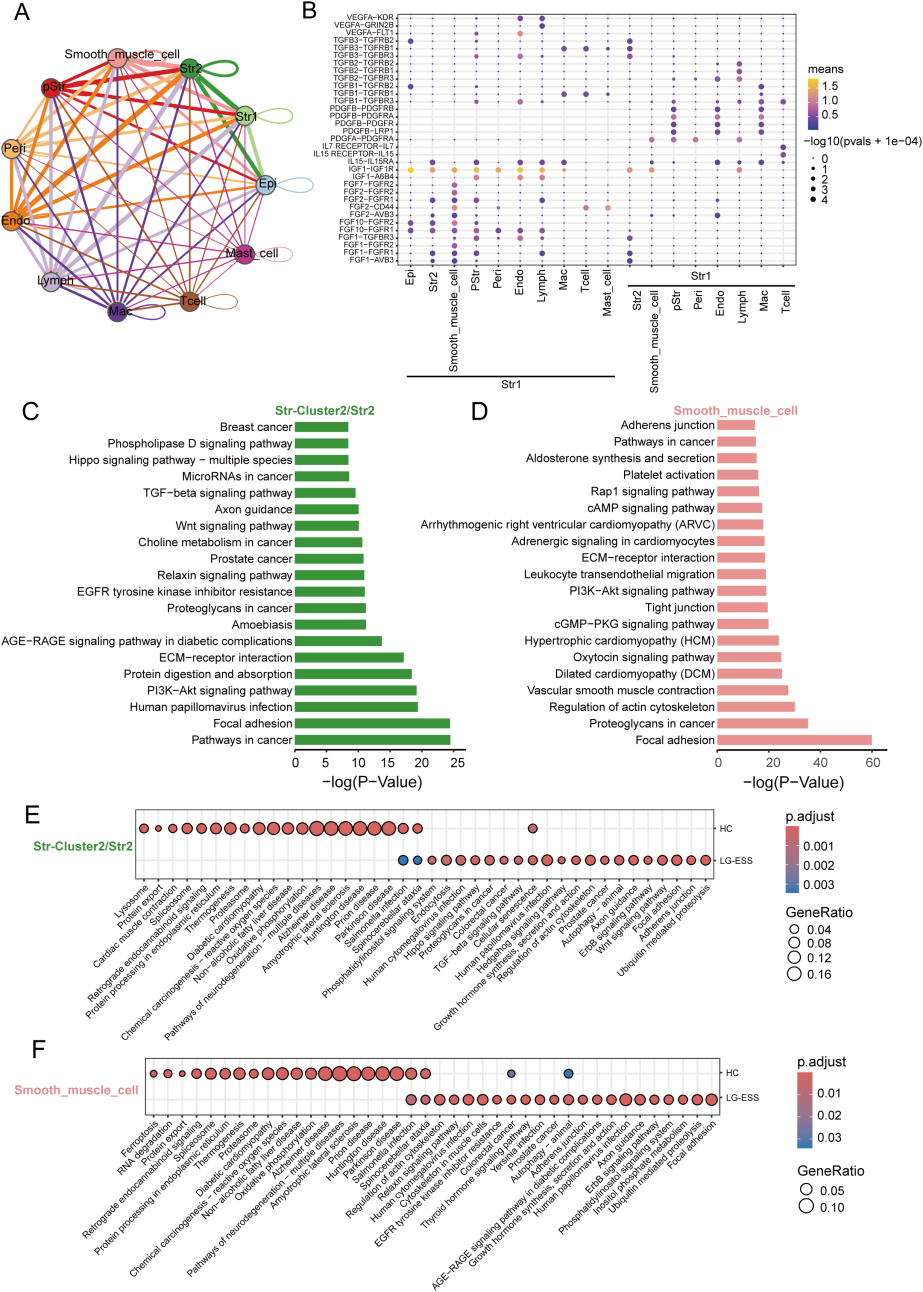
The functions of the Str2 and smooth muscle cells in the LG-ESS were altered. **(A)** Intercellular communication capacity between different cell types in LG-ESS. The color and thickness of the lines are used to distinguish the cellular origin and number of interacting ligands, and the circular lines indicate autocrine circuits. **(B)** Scatterplot indicating the interaction between Str1 and other cell subpopulations in LG-ESS via growth factor ligand-receptors. **(C, D)** Results of KEGG enrichment analysis of genes highly expressed in Str2 **(C)** and smooth muscle cells **(D)** in LG-ESS. **(E, F)** Scatter plots showing the results of KEGG enrichment analysis of differentially expressed genes in Str2 **(E)** and smooth muscle cells **(F)** in LG-ESS and compared to HC.

Through intercellular communication analysis, we identified a strong interaction between Str1 and Str2 as well as smooth muscle cell subpopulations in LG-ESS, particularly mediated by secreted growth factors (**Figures 5A, B**). We hypothesize that these interactions may lead to functional alterations in the subpopulations, thereby promoting tumor progression. To further explore these potential functional changes, we performed KEGG enrichment analysis on the highly expressed genes in the Str2 and smooth muscle cell subpopulations in LG-ESS. Interestingly, the genes predominantly clustered in pathways associated with tumorigenesis and metastasis, such as focal adhesion, proteoglycans in cancer, PI3K-Akt signaling, and ECM-receptor interactions (**Figures 5C, D**). Differential gene expression analysis between LG-ESS and healthy controls further supported these findings, indicating that the functions of Str2 and smooth muscle cells in LG-ESS are altered under the influence of tumor cells (**Figures 5E, F**).

## Discussion

LG-ESS is a rare uterine malignancy with a high recurrence rate, posing significant challenges for prognosis and treatment. Understanding the molecular characteristics and identifying prognostic biomarkers for LG-ESS is crucial for improving patient stratification and guiding personalized treatment. In this study, we used scRNA-seq to uncover the molecular characterization of tumor cell subpopulations within LG-ESS and their association with clinical outcomes. Our primary goal was to explore potential biomarkers linked to poor prognosis, offering new insights into disease progression.

Through the integration of single-cell transcriptomic data from LG-ESS patients and HC, we delineated 12 major cell subpopulations, highlighting the complexity of the tumor microenvironment and its potential role in the pathogenesis of LG-ESS. Among these subpopulations, the Str in particular displayed substantial gene expression changes, underscoring their involvement in LG-ESS tumorigenesis. The identification of two distinct stromal subgroups, Str1 (MME^+^ tumor cells) and Str2 (MME^-^ stromal fibroblasts), through reclustering of the Str subpopulation, has shed light on their divergent roles in LG-ESS. Our KEGG enrichment analysis revealed that Str1 is significantly enriched in pathways associated with oncogenesis, including Wnt signaling, focal adhesion, and PI3K-Akt signaling. The association between the abundance of Str1 and poor patient prognosis further accentuates the clinical relevance of this subpopulation. This suggests that Str1, characterized by their expression of MME, may play a central role in the malignant transformation and progression of LG-ESS.

Using RNA velocity analysis, we demonstrated that Str1 and Str2 cells share a common ancestral origin, suggesting that the differentiation of Str1 from normal stromal fibroblasts may be a key event in LG-ESS pathogenesis. This finding contradicts our initial hypothesis that Str1 arose directly from Str2, instead indicating a more complex tumorigenic process. Our pseudo-time analysis further confirmed that Str1 cells develop from an ancestral fibroblast population, highlighting the dynamic nature of tumor evolution in LG-ESS. Functional analysis of the gene expression changes during the differentiation of Str1 tumor cells further demonstrated the activation of key oncogenic pathways. Genes that were upregulated during the progression from normal fibroblasts to Str1 cells were enriched in pathways linked to cancer cell migration, proliferation, and extracellular matrix remodeling, all of which are hallmarks of tumor progression. Conversely, the downregulation of metabolic pathways, such as oxidative phosphorylation, suggests that tumor cells may undergo metabolic reprogramming as they acquire malignant characteristics. These observations are consistent with the metabolic changes often seen in cancer cells, which shift from oxidative phosphorylation to glycolysis to support rapid proliferation and survival in hypoxic environments.

Previous studies have indicated that the Wnt signaling pathway is significantly activated in LG-ESS (21, 38). Additionally, other research has found that FGF signaling plays a crucial role in Wnt-regulated cell proliferation (42), with FGF inhibition negating Wnt-mediated cell proliferation (43). In our study, *FGF12* and *KLHL29* were found to be significantly overexpressed in Str1 cells, and their high expression levels were associated with shorter overall survival and progression-free survival. It is worth noting that the overexpression of *FGF12* was also observed in an independent cohort of uterine sarcoma patients and was significantly associated with a decrease in patient survival, further confirming its prognostic significance.

While *FGF12* serves as a prognostic biomarker associated with poor outcomes in LG-ESS, *KLHL29* also emerged as a potentially significant marker in this context. Our analysis revealed that *KLHL29* expression levels vary across different sarcoma subtypes, which may help distinguish between high-grade and low-grade ESS and LMS. This differential expression suggests that *KLHL29* could be valuable for clinical stratification of ESS patients, providing insights into tumor behavior and treatment responses. While *FGF12* may reflect the aggressiveness of the tumor microenvironment, *KLHL29*’s varying expression levels among different sarcoma types highlight its potential as a biomarker for distinguishing tumor subtypes. The synergistic evaluation of both *FGF12* and *KLHL29* may enhance our understanding of LG-ESS progression and guide more tailored therapeutic strategies.

In tumors, FGF is essential for maintaining endothelial integrity, promoting angiogenesis, and supporting tumor proliferation, survival, and metastasis (44, 45). Abnormal FGF signaling accelerates tumor growth by enhancing the formation of new blood vessels, which makes FGF inhibitors a promising therapeutic strategy (46–48). The association between the tumor cell subpopulation Str1 and its marker *FGF12* with poor prognosis in ESS suggests that targeting the FGF signaling pathway could be a promising therapeutic approach. Furthermore, their abundance/expression levels show no significant differences across different ESS subtypes, indicating that despite the clinical variations among ESS subtypes, they may share some key molecular characteristics that lead to similar prognostic outcomes. The relative stability of Str1 and *FGF12* may reflect their importance within the tumor microenvironment, particularly in promoting tumor cell survival and proliferation. Therefore, specific *FGF12*-targeted therapies, including selective FGFR inhibitors and neutralizing antibodies, could potentially be developed to inhibit FGF-mediated cell proliferation across all ESS subtypes. FGF receptor inhibitors, such as FGFR tyrosine kinase inhibitors (49), have shown efficacy in various cancers with aberrant FGF signaling and may represent a potential treatment avenue for ESS patients with high *FGF12* expression. However, developing FGF inhibitors presents certain challenges, as the FGF/FGFR signaling axis is vital for many normal biological processes, raising concerns about potential toxicity and necessitating careful dosage management (48). Furthermore, overcoming resistance may require combining FGF inhibitors with other therapeutic agents to enhance treatment efficacy (50).

The functional consequences of *FGF12* and *KLHL29* overexpression were further explored through gene set enrichment analysis (GSEA). Our results showed that high expression of both genes was associated with the activation of the MYOGENESIS pathway, a process often linked to tissue remodeling and cancer progression. In contrast, immune-related pathways such as the inflammatory response and interferon-gamma response were suppressed in the *FGF12* ^high^ group, implying that *FGF12* may contribute to immune evasion in LG-ESS. Similarly, *KLHL29* ^high^ tumors exhibited downregulation of the estrogen response early pathway, which may reflect an altered hormonal milieu in these tumors. This suggests that *FGF12* and *KLHL29* may play functional roles in promoting LG-ESS progression through different biological pathways.

Our study also emphasizes the broader impact of tumor cells on the surrounding stromal and immune compartments within the LG-ESS microenvironment. We observed strong intercellular interactions between Str1 tumor cells and smooth muscle cell subpopulations, particularly through growth factor-mediated signaling. These interactions are likely to drive functional changes in both stromal and smooth muscle cells, as evidenced by our KEGG analysis, which revealed enrichment of genes related to tumorigenesis and metastasis in these cell types. This indicates that stromal fibroblasts and smooth muscle cells within the tumor microenvironment can undergo phenotypic and functional alterations that promote tumor growth and invasion. The activation of CAFs and M2 macrophage polarization in epithelial tumors serves as a parallel example of how tumor cells can reshape their surrounding microenvironment to facilitate malignancy. Our findings suggest that a similar mechanism may be at play in LG-ESS, where tumor-derived factors drive the reprogramming of stromal and immune cells, ultimately contributing to disease progression.

While our study provides valuable insights into the biology of LG-ESS, several limitations should be acknowledged. First, given the rarity of LG-ESS, our single-cell RNA sequencing analysis was conducted on a sample from one LG-ESS patient. Although this single-sample design constrains generalizability, we addressed this limitation by integrating publicly available transcriptomic datasets and validating key findings—such as the prognostic significance of *FGF12*—within independent cohorts. These steps strengthen the reliability of our observations, yet additional studies with larger patient cohorts are essential to validate our findings and to capture the full spectrum of LG-ESS heterogeneity. Additionally, our study identified the tumor cell subgroup Str1, characterized by MME^+^ (gene of CD10 protein) expression, through a combination of H&E and miF staining. A minor population of cells in the Str2 subgroup also exhibited diffuse CD10 expression. However, since CD10 is expressed in both normal endometrial stromal cells and LG-ESS tumor cells (36, 37), CD10 alone cannot reliably distinguish malignant cells. This suggests that while some Str2 cells may have undergone malignant transformation, further marker studies are required to confirm their tumorigenic nature. Moreover, while we focused on stromal fibroblasts and their role in tumor progression, other cellular components of the tumor microenvironment, such as immune cells, were not analyzed in depth. Given the importance of immune-stromal crosstalk in tumor biology, further exploration of the interactions between tumor cells, stromal fibroblasts, and immune cells will be critical.

In conclusion, our study highlights the heterogeneity of stromal fibroblasts in LG-ESS and their critical role in tumor progression. The identification of Str1 as a poor prognostic factor and the discovery of *FGF12* as a prognostic biomarker opens new avenues for understanding and treating this rare form of uterine sarcoma.

## Data Availability

The datasets presented in this study can be found in online repositories. The names of the repository/repositories and accession number(s) can be found below: https://zenodo.org/records/, 13946278.

## Supplementary material

Supplementary Figure 1 Results of removing batch effects for data from different sources and number of cells in all subpopulations. **(A)** Scatterplot plot showing the well-corrected batch effect (harmony) of different sources of HC and LG-ESS patient data. **(B)** Heatmap showing the expression levels of highly expressed top5 genes in all subpopulations. **(C)** The table shows the number of cells from all samples in each subpopulation.

Supplementary Figure 2 Elevated Proportion of Str1 in ESS Correlates with Reduced Survival. **(A)** miF staining result of LG-ESS sample. CD10, desmin,α-SMA, and other markers are used to distinguish different cell populations within the tumor, with magnified views focusing on specific regions of interest. The scale bars represent 100µm and 30µm. **(B)** Survival analysis results of the proportion of Str1 in ESS patients, with the left panel representing overall survival and the right panel depicting progression-free survival (GSE128630). **(C)** Variations in the proportion of Str1 among different subtypes of uterine sarcoma patients (GSE85383). (Wilcoxon rank-sum test; ns, non-significant)

Supplementary Figure 3
*FGF12* has potential as a prognostic biomarker for sarcoma. **(A, B)** Multivariate Cox regression analysis for overall survival **(A)** and progression-free survival **(B)** in LG-ESS patients. The analysis included relevant clinical factors, such as age at diagnosis, tumor grade, and BMI, alongside the expression levels of *FGF12* and *KLHL29*. Hazard ratios (HR) and 95% confidence intervals (CI) are shown for each factor. **(C)** Survival analysis outcomes based on the expression of *FGF12* (left) and *KLHL29* (right) genes in uterine sarcoma patients (GSE119041), focusing on overall survival. **(D)** Differential expression of *FGF12* and *KLHL29* genes among various subtypes of uterine sarcoma patients. (Wilcoxon rank-sum test; **P* < 0.05; ***P* < 0.01; ns, non-significant)

## References

[1] Stewart LE Beck TL Giannakopoulos NV Rendi MH Isacson C Goff BA. Impact of oophorectomy and hormone suppression in low grade endometrial stromal sarcoma: A multicenter review. Gynecol Oncol. (2018) 149(2):297–300. doi: 10.1016/j.ygyno.2018.03.008 29534832

[2] Rauh-Hain JA Del Carmen MG. Endometrial stromal sarcoma: a systematic review. Obstet Gynecol. (2013) 122(3):676–83. doi: 10.1097/AOG.0b013e3182a189ac 23921879

[3] Höhn AK Brambs CE Hiller GGR May D Schmoeckel E Horn LC. 2020 WHO classification of female genital tumors. Geburtshilfe Frauenheilkd. (2021) 81(10):1145–53. doi: 10.1055/a-1545-4279 PMC8494521 34629493

[4] Gadducci A Multinu F De Vitis LA Cosio S Carinelli S Aletti GD. Endometrial stromal tumors of the uterus: Epidemiology, pathological and biological features, treatment options and clinical outcomes. Gynecol Oncol. (2023) 171:95–105. doi: 10.1016/j.ygyno.2023.02.009 36842409

[5] Kikuchi A Yoshida H Tsuda H Nishio S Suzuki S Takehara K. Clinical characteristics and prognostic factors of endometrial stromal sarcoma and undifferentiated uterine sarcoma confirmed by central pathologic review: A multi-institutional retrospective study from the Japanese Clinical Oncology Group. Gynecol Oncol. (2023) 176:82–9. doi: 10.1016/j.ygyno.2023.07.002 37478616

[6] Desar IME Ottevanger PB Benson C van der Graaf WTA. Systemic treatment in adult uterine sarcomas. Crit Rev Oncol Hematol. (2018) 122:10–20. doi: 10.1016/j.critrevonc.2017.12.009 29458779

[7] Dessources K Miller KM Kertowidjojo E Da Cruz Paula A Zou Y Selenica P. ESR1 hotspot mutations in endometrial stromal sarcoma with high-grade transformation and endocrine treatment. Mod Pathol. (2022) 35(7):972–8. doi: 10.1038/s41379-021-01003-5 PMC9234101 34961764

[8] Chu MC Mor G Lim C Zheng W Parkash V Schwartz PE. Low-grade endometrial stromal sarcoma: hormonal aspects. Gynecol Oncol. (2003) 90(1):170–6. doi: 10.1016/S0090-8258(03)00258-0 12821359

[9] Yamaguchi M Erdenebaatar C Saito F Motohara T Miyahara Y Tashiro H. Long-term outcome of aromatase inhibitor therapy with letrozole in patients with advanced low-grade endometrial stromal sarcoma. Int J Gynecol Cancer. (2015) 25(9):1645–51. doi: 10.1097/IGC.0000000000000557 26495759

[10] Yamamoto M Tsujikawa T Yamada S Kurokawa T Shinagawa A Chino Y. 18F-FDG/18F-FES standardized uptake value ratio determined using PET predicts prognosis in uterine sarcoma. Oncotarget. (2017) 8(14):22581–9. doi: 10.18632/oncotarget.15127 PMC5410246 28186981

[11] Zhao Z Yoshida Y Kurokawa T Kiyono Y Mori T Okazawa H. 18F-FES and 18F-FDG PET for differential diagnosis and quantitative evaluation of mesenchymal uterine tumors: correlation with immunohistochemical analysis. J Nucl Med. (2013) 54(4):499–506. doi: 10.2967/jnumed.112.113472 23471314

[12] Ali SI Sayyed RH Bakar MA Sadaf T Syed AA. A retrospective study of endometrial stromal sarcoma: an institutional review. J Pak Med Assoc. (2020) 70(5):926–9. doi: 10.5455/JPMA.299808 32400756

[13] Bai H Yang J Cao D Huang H Xiang Y Wu M. Ovary and uterus-sparing procedures for low-grade endometrial stromal sarcoma: a retrospective study of 153 cases. Gynecol Oncol. (2014) 132(3):654–60. doi: 10.1016/j.ygyno.2013.12.032 24412112

[14] Wu J Zhang H Li L Hu M Chen L Xu B. A nomogram for predicting overall survival in patients with low-grade endometrial stromal sarcoma: A population-based analysis. Cancer Commun (Lond). (2020) 40(7):301–12. doi: 10.1002/cac2.12067 PMC7365459 32558385

[15] Seagle BL Shilpi A Buchanan S Goodman C Shahabi S. Low-grade and high-grade endometrial stromal sarcoma: A National Cancer Database study. Gynecol Oncol. (2017) 146(2):254–62. doi: 10.1016/j.ygyno.2017.05.036 28596015

[16] Barney B Tward JD Skidmore T Gaffney DK. Does radiotherapy or lymphadenectomy improve survival in endometrial stromal sarcoma? Int J Gynecol Cancer. (2009) 19(7):1232–8. doi: 10.1111/IGC.0b013e3181b33c9a 19823060

[17] Kang N Zhang Y Guo S Chen R Kong F Wang S. Genomic and transcriptomic characterization revealed the high sensitivity of targeted therapy and immunotherapy in a subset of endometrial stromal sarcoma. Cancer Res Treat. (2023) 55(3):978–91. doi: 10.4143/crt.2022.1647 PMC10372608 36731460

[18] Sylvestre VT Dunton CJ. Treatment of recurrent endometrial stromal sarcoma with letrozole: a case report and literature review. Horm Cancer. (2010) 1(2):112–5. doi: 10.1007/s12672-010-0007-9 PMC10358008 21761354

[19] Conklin CM Longacre TA. Endometrial stromal tumors: the new WHO classification. Adv Anat Pathol. (2014) 21(6):383–93. doi: 10.1097/PAP.0000000000000046 25299308

[20] Kim GW Baek SK Han JJ Kim HJ Sung JY Maeng CH. Pulmonary metastasizing low-grade endometrial stromal sarcoma: case report and review of diagnostic pitfalls. Diagnost (Basel). (2022) 12(2). doi: 10.3390/diagnostics12020271 PMC8871004 35204363

[21] Micci F Heim S Panagopoulos I. Molecular pathogenesis and prognostication of "low-grade'' and "high-grade" endometrial stromal sarcoma. Genes Chromosomes Cancer. (2021) 60(3):160–7. doi: 10.1002/gcc.22907 PMC7894482 33099834

[22] Miao Y Konno Y Wang B Zhu L Zhai T Ihira K. Integrated multi-omics analyses and functional validation reveal TTK as a novel EMT activator for endometrial cancer. J Transl Med. (2023) 21(1):151. doi: 10.1186/s12967-023-03998-8 36829176 PMC9960418

[23] Ren X Liang J Zhang Y Jiang N Xu Y Qiu M. Single-cell transcriptomic analysis highlights origin and pathological process of human endometrioid endometrial carcinoma. Nat Commun. (2022) 13(1):6300. doi: 10.1038/s41467-022-33982-7 36273006 PMC9588071

[24] Gotoh O Sugiyama Y Takazawa Y Kato K Tanaka N Omatsu K. Clinically relevant molecular subtypes and genomic alteration-independent differentiation in gynecologic carcinosarcoma. Nat Commun. (2019) 10(1):4965. doi: 10.1038/s41467-019-12985-x 31672974 PMC6823358

[25] Gultekin O Gonzalez-Molina J Hardell E Moyano-Galceran L Mitsios N Mulder J. FOXP3+ T cells in uterine sarcomas are associated with favorable prognosis, low extracellular matrix expression and reduced YAP activation. NPJ Precis Oncol. (2021) 5(1):97. doi: 10.1038/s41698-021-00236-6 34799669 PMC8604926

[26] Przybyl J Kowalewska M Quattrone A Dewaele B Vanspauwen V Varma S. Macrophage infiltration and genetic landscape of undifferentiated uterine sarcomas. JCI Insight. (2017) 2(11). doi: 10.1172/jci.insight.94033 PMC5453711 28570276

[27] Mcginnis CS Murrow LM Gartner ZJ. DoubletFinder: doublet detection in single-cell RNA sequencing data using artificial nearest neighbors. Cell Syst. (2019) 8(4):329–37.e4. doi: 10.1016/j.cels.2019.03.003 30954475 PMC6853612

[28] Hao Y Hao S Andersen-Nissen E Mauck WM Zheng S Butler A. Integrated analysis of multimodal single-cell data. Cell. (2021) 184(13). doi: 10.1016/j.cell.2021.04.048 PMC8238499 34062119

[29] Korsunsky I Millard N Fan J Slowikowski K Zhang F Wei K. Fast, sensitive and accurate integration of single-cell data with Harmony. Nat Methods. (2019) 16(12):1289–96. doi: 10.1038/s41592-019-0619-0 PMC6884693 31740819

[30] Lv H Zhao G Jiang P Wang H Wang Z Yao S. Deciphering the endometrial niche of human thin endometrium at single-cell resolution. Proc Natl Acad Sci U.S.A. (2022) 119(8). doi: 10.1073/pnas.2115912119 PMC8872762 35169075

[31] La Manno G Soldatov R Zeisel A Braun E Hochgerner H Petukhov V. RNA velocity of single cells. Nature. (2018) 560(7719):494–8. doi: 10.1038/s41586-018-0414-6 PMC6130801 30089906

[32] Qiu X Mao Q Tang Y Wang L Chawla R Pliner HA. Reversed graph embedding resolves complex single-cell trajectories. Nat Methods. (2017) 14(10):979–82. doi: 10.1038/nmeth.4402 PMC5764547 28825705

[33] Efremova M Vento-Tormo M Teichmann SA Vento-Tormo R. CellPhoneDB: inferring cell-cell communication from combined expression of multi-subunit ligand-receptor complexes. Nat Protoc. (2020) 15(4):1484–506. doi: 10.1038/s41596-020-0292-x 32103204

[34] Jew B Alvarez M Rahmani E Miao Z Ko A Garske KM. Accurate estimation of cell composition in bulk expression through robust integration of single-cell information. Nat Commun. (2020) 11(1):1971. doi: 10.1038/s41467-020-15816-6 32332754 PMC7181686

[35] Yu G Wang LG Han Y He QY. clusterProfiler: an R package for comparing biological themes among gene clusters. Omics. (2012) 16(5):284–7. doi: 10.1089/omi.2011.0118 PMC3339379 22455463

[36] Mccluggage WG Sumathi VP Maxwell P. CD10 is a sensitive and diagnostically useful immunohistochemical marker of normal endometrial stroma and of endometrial stromal neoplasms. Histopathology. (2001) 39(3):273–8. doi: 10.1046/j.1365-2559.2001.01215.x 11532038

[37] Akaev I Yeoh CC Rahimi S. Update on endometrial stromal tumours of the uterus. Diagnost (Basel). (2021) 11(3). doi: 10.3390/diagnostics11030429 PMC8000701 33802452

[38] Przybyl J Kidzinski L Hastie T Debiec-Rychter M Nusse R van de Rijn M. Gene expression profiling of low-grade endometrial stromal sarcoma indicates fusion protein-mediated activation of the Wnt signaling pathway. Gynecol Oncol. (2018) 149(2):388–93. doi: 10.1016/j.ygyno.2018.03.007 29544705

[39] Mao X Xu J Wang W Liang C Hua J Liu J. Crosstalk between cancer-associated fibroblasts and immune cells in the tumor microenvironment: new findings and future perspectives. Mol Cancer. (2021) 20(1):131. doi: 10.1186/s12943-021-01428-1 34635121 PMC8504100

[40] Kennel KB Bozlar M De Valk AF Greten FR. Cancer-associated fibroblasts in inflammation and antitumor immunity. Clin Cancer Res. (2023) 29(6):1009–16. doi: 10.1158/1078-0432.CCR-22-1031 PMC10011884 36399325

[41] Zhao S Mi Y Guan B Zheng B Wei P Gu Y. Tumor-derived exosomal miR-934 induces macrophage M2 polarization to promote liver metastasis of colorectal cancer. J Hematol Oncol. (2020) 13(1):156. doi: 10.1186/s13045-020-00991-2 33213490 PMC7678301

[42] Ten Berge D Brugmann SA Helms JA Nusse R. Wnt and FGF signals interact to coordinate growth with cell fate specification during limb development. Development. (2008) 135(19):3247–57. doi: 10.1242/dev.023176 PMC2756806 18776145

[43] Tang D He Y Li W Li H. Wnt/β-catenin interacts with the FGF pathway to promote proliferation and regenerative cell proliferation in the zebrafish lateral line neuromast. Exp Mol Med. (2019) 51(5):1–16. doi: 10.1038/s12276-019-0247-x PMC6533250 31123246

[44] Zhou Y Tao L Qiu J Xu J Yang X Zhang Y. Tumor biomarkers for diagnosis, prognosis and targeted therapy. Signal Transduct Target Ther. (2024) 9(1):132. doi: 10.1038/s41392-024-01823-2 38763973 PMC11102923

[45] Presta M Dell'era P Mitola S Moroni E Ronca R Rusnati M. Fibroblast growth factor/fibroblast growth factor receptor system in angiogenesis. Cytokine Growth Factor Rev. (2005) 16(2):159–78. doi: 10.1016/j.cytogfr.2005.01.004 15863032

[46] Mashreghi M Azarpara H Bazaz MR Jafari A Masoudifar A Mirzaei H. Angiogenesis biomarkers and their targeting ligands as potential targets for tumor angiogenesis. J Cell Physiol. (2018) 233(4):2949–65. doi: 10.1002/jcp.v233.4 28608549

[47] Yashiro M Matsuoka T. Fibroblast growth factor receptor signaling as therapeutic targets in gastric cancer. World J Gastroenterol. (2016) 22(8):2415–23. doi: 10.3748/wjg.v22.i8.2415 PMC4768188 26937130

[48] Brooks AN Kilgour E Smith PD. Molecular pathways: fibroblast growth factor signaling: a new therapeutic opportunity in cancer. Clin Cancer Res. (2012) 18(7):1855–62. doi: 10.1158/1078-0432.CCR-11-0699 22388515

[49] Xie Y Su N Yang J Tan Q Huang S Jin M. FGF/FGFR signaling in health and disease. Signal Transduct Target Ther. (2020) 5(1):181. doi: 10.1038/s41392-020-00222-7 32879300 PMC7468161

[50] Dieci MV Arnedos M Andre F Soria JC. Fibroblast growth factor receptor inhibitors as a cancer treatment: from a biologic rationale to medical perspectives. Cancer Discovery. (2013) 3(3):264–79. doi: 10.1158/2159-8290.CD-12-0362 23418312

