# Supplementary figures and images for: Single-cell RNA-seq reveals FGF12 as a prognostic biomarker in low-grade endometrial stromal sarcoma
Source: Front Immunol. 2024 Nov 29;15:1513076. doi: 10.3389/fimmu.2024.1513076 (PMC11638184; doi:10.3389/fimmu.2024.1513076)

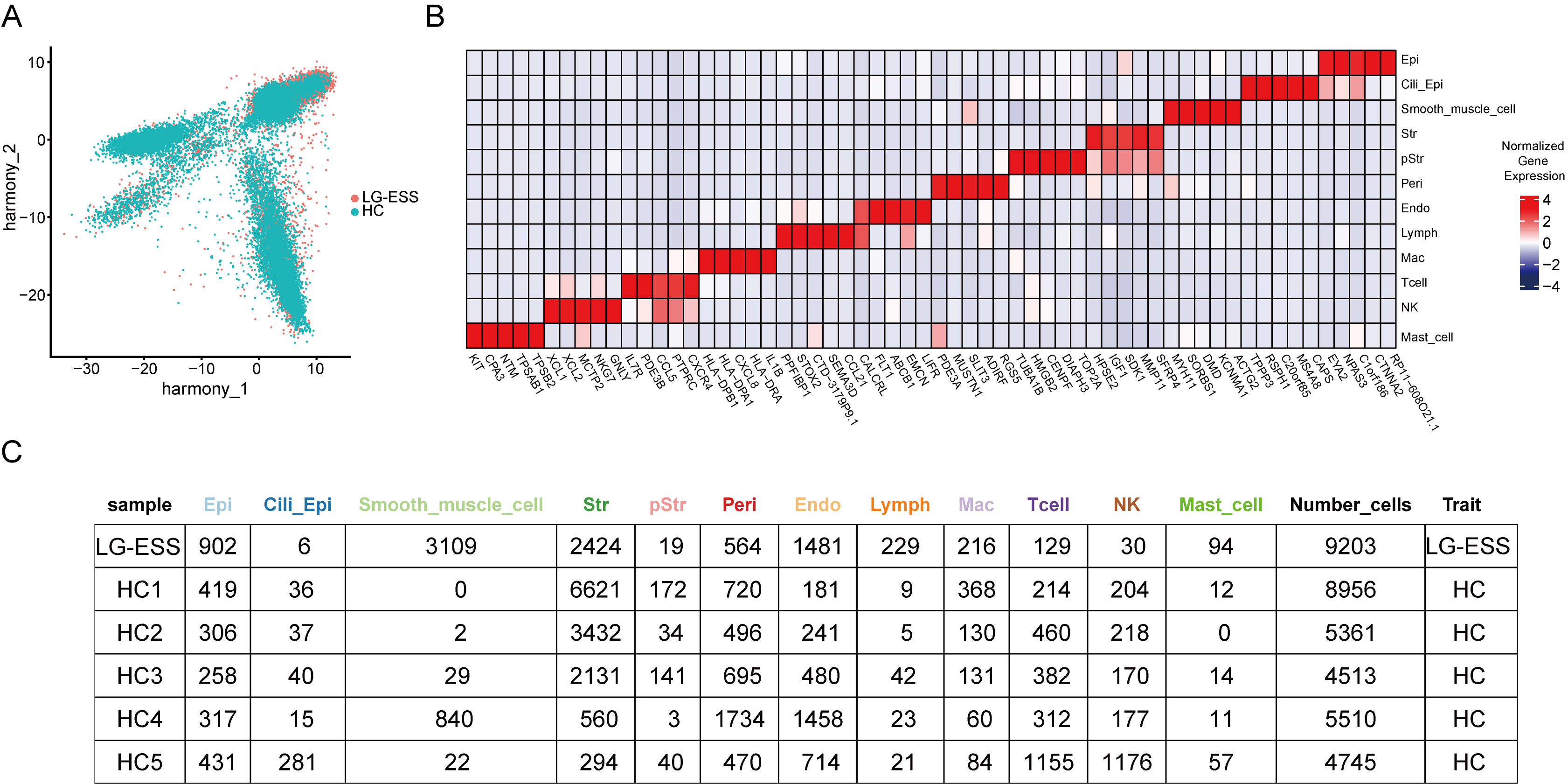

Supplement: Supplementary Figure 1 — Results of removing batch effects for data from different sources and number of cells in all subpopulations. (A) Scatterplot plot showing the well-corrected batch effect (harmony) of different sources of HC and LG-ESS patient data. (B) Heatmap showing the expression levels of highly expressed top5 genes in all subpopulations. (C) The table shows the number of cells from all samples in each subpopulation. [file Image1.tif]

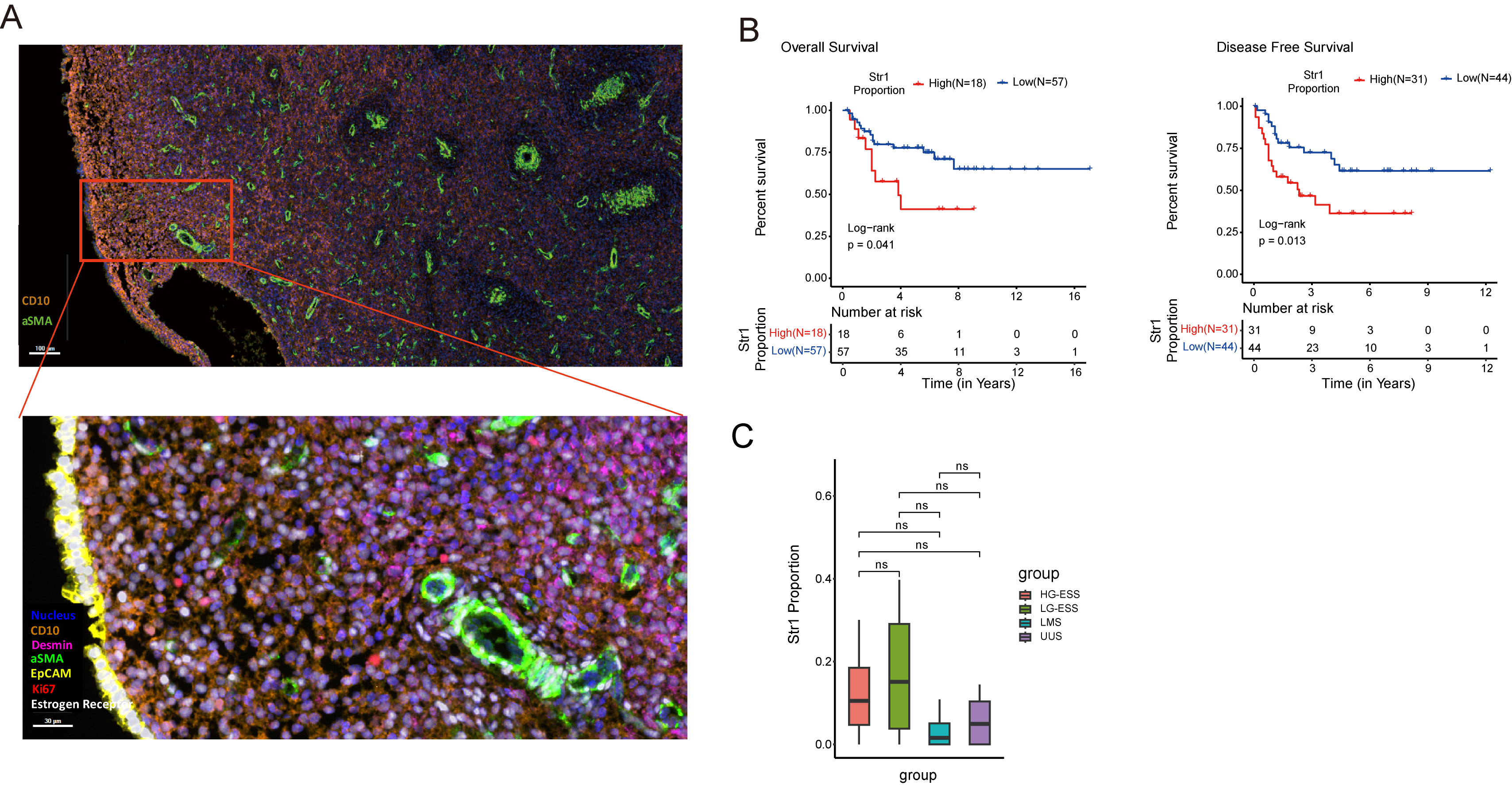

Supplement: Supplementary Figure 2 — Elevated Proportion of Str1 in ESS Correlates with Reduced Survival. (A) miF staining result of LG-ESS sample. CD10, desmin,α-SMA, and other markers are used to distinguish different cell populations within the tumor, with magnified views focusing on specific regions of interest. The scale bars represent 100µm and 30µm. (B) Survival analysis results of the proportion of Str1 in ESS patients, with the left panel representing overall survival and the right panel depicting progression-free survival (GSE128630). (C) Variations in the proportion of Str1 among different subtypes of uterine sarcoma patients (GSE85383). (Wilcoxon rank-sum test; ns, non-significant) [file Image2.tif]

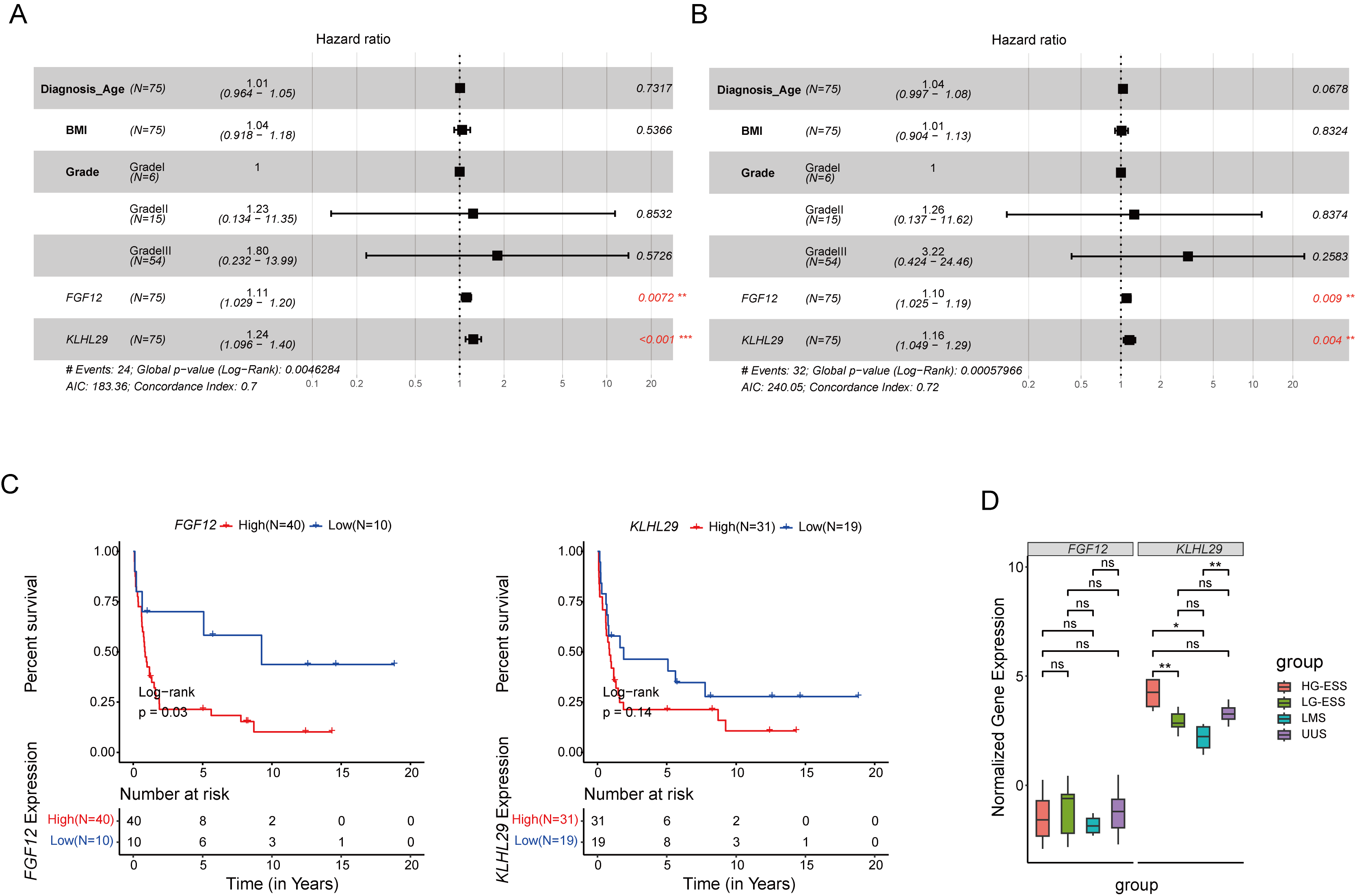

Supplement: Supplementary Figure 3 — FGF12 has potential as a prognostic biomarker for sarcoma. (A, B) Multivariate Cox regression analysis for overall survival (A) and progression-free survival (B) in LG-ESS patients. The analysis included relevant clinical factors, such as age at diagnosis, tumor grade, and BMI, alongside the expression levels of FGF12 and KLHL29. Hazard ratios (HR) and 95% confidence intervals (CI) are shown for each factor. (C) Survival analysis outcomes based on the expression of FGF12 (left) and KLHL29 (right) genes in uterine sarcoma patients (GSE119041), focusing on overall survival. (D) Differential expression of FGF12 and KLHL29 genes among various subtypes of uterine sarcoma patients. (Wilcoxon rank-sum test; *P < 0.05; **P < 0.01; ns, non-significant) [file Image3.tif]
